# IS FRAILTY THE END? ASSOCIATION OF LIFESTYLES AND SOCIAL ENVIRONMENT WITH DEATH AMONG THE FRAIL

**DOI:** 10.1093/geroni/igad104.1448

**Published:** 2023-12-21

## Abstract

Despite many efforts, reversing the frail status among older adults remains difficult. However, frailty does not mean the end of life. Adherence to a healthy lifestyle and a desirable social environment may reduce the risk of adverse outcomes among the frail. We examined whether adherence to a healthy lifestyle was associated with lower all-cause mortality and the joint association of lifestyles and social environment with mortality among the frail. Data were from UK Biobank; 15,594 physically frail middle-aged and older adults were included. Frailty was assessed by Fried’s physical frailty phenotype approach. We created a composite healthy lifestyle score using four lifestyle factors: smoking, alcohol consumption, physical activity, and diet. We used 17 social factors to construct a polysocial score for measuring the social environment. We used the Cox model to estimate the association of each lifestyle factor and the composite lifestyle score with mortality among the entire sample and by polysocial score (low, intermediate, and high level). We also evaluated the joint association of lifestyle and polysocial scores with mortality. Frail participants with a healthy level of smoking, physical activity, diet, and composite lifestyle score had a 40%, 33%, 15%, and 34% lower hazard of death than those with an unhealthy level, respectively. We found a joint effect of a healthy lifestyle and social environment on mortality. Adherence to a healthy lifestyle may provide an opportunity to decrease the risk of adverse health outcomes among the frail, especially among those living in an unfavorable social environment.

